# Long-term trajectories of BMI predict carotid stiffness and plaque volume in type 2 diabetes older adults: a cohort study

**DOI:** 10.1186/s12933-020-01104-6

**Published:** 2020-09-15

**Authors:** Chen Botvin Moshe, Salo Haratz, Ramit Ravona-Springer, Anthony Heymann, Lin Hung-Mo, Michal Schnaider Beeri, David Tanne

**Affiliations:** 1 The Joseph Sagol Neuroscience Center, Sheba Medical Center, Sackler School of Medicine, Tel Aviv University, Harimon 9, POB 365, 4295400 Nordia, Israel; 2 Assuta medical center, Ashdod, Israel; 3 Memory and Geriatric Psychiatry Clinic, Sheba Medical center, Sackler School of Medicine, Tel Aviv University, Tel Aviv-Yafo, Israel; 4 Maccabi Health Services, Israel, Sackler School of Medicine, Tel Aviv University, Tel Aviv-Yafo, Israel; 5 Department of Anesthesiology, Icahn School of Medicine at Mount Sinai, New York, USA; 6 The Joseph Sagol Neuroscience Center, Sheba Medical Center, Ramat Ga, Israel; 7 Department of Psychiatry, Icahn School of Medicine at Mount Sinai, New York, NY USA; 8 Stroke and Cognition Institute, Rambam Health Care Campus, Haifa, Israel

**Keywords:** Obesity, Diabetes, Carotid Atherosclerosis

## Abstract

**Background:**

High body mass index (BMI) is a risk factor for type 2 diabetes and cardiovascular disease. However, its relationships with indices of carotid stiffness and plaque volume are unclear. We investigated associations of long-term measurements of BMI with indices of carotid stiffness and atherosclerosis among non-demented diabetes patients from the Israel Diabetes and Cognitive Decline (IDCD) study.

**Methods:**

Carotid ultrasound indices [carotid intima media thickness (cIMT), distensibility, elastography and plaque volume] were assessed in N = 471 participants. Mean BMI across all MHS diabetes registry measurements and trajectories of BMI were calculated. BMI was categorized into three trajectory groups representing: a relatively stable normal weight (n = 185, 44%), overweight trajectory (n = 188, 44.8%) and a trajectory of obesity (n = 47, 11.2%). Linear and logistic regressions estimated associations of carotid indices with mean BMI and BMI trajectories.

**Results:**

Compared to the normal weight trajectory, an obesity trajectory was associated with carotid distensibility (β = − 3.078, p = 0.037), cIMT (β = 0.095, p = 0.004), and carotid elastography (β = 0.181, p = 0.004) but not with plaque volume (β = 0.066, p = 0.858). Compared with the normal weight trajectory, an obesity trajectory was associated with increased odds for impaired carotid distensibility (OR = 2.790, p = 0.033), impaired cIMT (OR = 5.277, p = 0.001) and large carotid plaque volume (OR = 8.456, p = 0.013) but not with carotid elastography (OR = 1.956, p = 0.140). Mean BMI was linearly associated with Distensibility (β = − 0.275, p = 0.005) and cIMT (β = 0.005, p = 0.026).

**Conclusions:**

Long-term measurements of adiposity are associated with indices of carotid stiffness and plaque volume among older type 2 diabetes adults.

## Background

Overweight and obesity, commonly measured by body mass index (BMI) [1, 2] have been shown to be an independent risk factor for type 2 diabetes, hypertension, cardiovascular disease and stroke [1]. In the United States nearly 35% of the adults are obese and obesity is the 5th leading cause of death [3]. The association of obesity with carotid atherosclerosis, as measured by cIMT and distensibility was demonstrated in several studies [4–7]. Vascular changes already develop among obese young children [8] and adolescents [9] suggesting that the exposure of obesity may affect vascular health throughout the life course.

Atherosclerosis is the underlying process of most cardiovascular disease. Carotid Intima Media Thickness (cIMT) was found to be a predictor for cardiovascular disease risk [4, 10], stroke [11], and all-cause mortality [12]. Recently some innovative ultrasound methods have been developed to assess pre-clinical markers of carotid atherosclerosis disease and to evaluate its progression: carotid artery distensibility and carotid shear-wave elastography. Carotid distensibility is a functional parameter that measures the arterial ability to expand and contract with cardiac pulsation and relaxation [13]. Functional impairment of the arterial wall may occur at an early stage of the atherosclerotic process before structural wall changes become detectable as well as before the occurrence of clinical symptoms of vascular disease [14]. Shear wave elastography of the carotid wall is an innovative method used to evaluate carotid artery wall stiffness. Elastography measures the structural property of the carotid artery wall and represents the artery tissue stiffness.

Despite the key role of obesity in the incidence of type 2 diabetes, and the consistently increased risk for carotid atherosclerosis in type 2 diabetes patients, evidence on the associations of long-term obesity with indices of carotid stiffness and atherosclerotic plaque volume in this high-risk population are scarce. In this study, we used data from the Israel Diabetes and Cognitive Decline (IDCD) study to investigate the association of long-term measurements of BMI with ultrasound indices of carotid arterial wall function and atherosclerotic plaque volume in cognitively normal type 2 diabetes older adults.

## Methods

### Study population

**Fig. 1:**
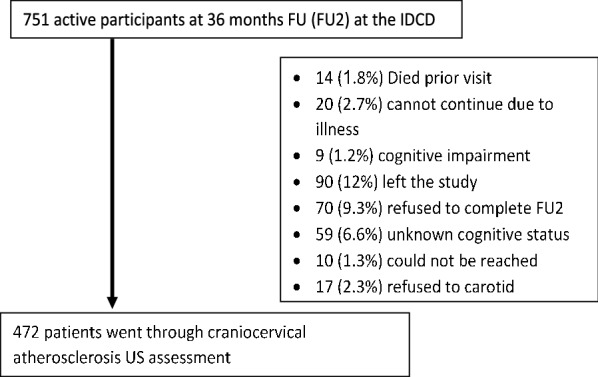
Flow chart of the patients in the IDCD carotid artery cohort

The IDCD study investigates the effects of long-term type 2 diabetes-related characteristics on cognitive decline and the study design has been previously described in detail [15]. Briefly, the IDCD recruited community-dwelling elderly individuals with type 2 diabetes (65 + years old) living in central Israel, from approximately 11,000 clients enrolled in the diabetes registry of the Maccabi Healthcare Services (MHS). MHS is the second largest health maintenance organization (HMO), treating a representative cross-section of 2 million citizens. The MHS diabetes registry was established in 1998 to facilitate diabetes management and to improve treatment to include patients according to criteria previously detailed [15]. IDCD inclusion criteria were having type 2 diabetes; normal cognition at entry; being free of any neurological (e.g., Parkinson’s disease, stroke), psychiatric (e.g., schizophrenia) or other diseases (e.g., alcohol or drug abuse) that might affect cognition; and having an informant. The IDCD cohort was established for the investigation of the contribution of T2D characteristics to cognitive decline among patients with type 2 diabetes. Therefore, the eligibility criteria were focused on factors that may be related to cognition rather than to global health. From the T2D angle, the goal was to include the broadest sample possible in terms of micro and macro complications, as those may themselves contribute to greater cognitive decline and dementia, so ketoacidosis, major hypoglycemia episodes, renal failure etc., were not exclusion criteria. Participants were assessed by a physician experienced in assessment and diagnosis of dementia, and by a neuropsychologist, who administered a broad neuropsychological battery. Four hundred and seventy-one (471) IDCD participants went through the carotid artery US assessments and had complete data on sociodemographic (Fig. 1). The study was approved by the Institutional Review Boards of all three institutions participating in the study (Ichan School of Medicine at Mount Sinai, NY, Sheba Medical Center, Israel and Maccabi Health Services, Israel) and all participants signed informed consent.

### Ultrasound assessment procedures

Carotid stiffness and atherosclerosis was assessed following the IDCD 36 months follow up visit. All examinations were performed at the Department of Neurology, Sheba Medical Center by one of 2 qualified and experienced ultrasound technicians, after obtaining informed consent. Subjects were placed in a supine position and rested for 5 min prior to assessing their vital signs. Carotid Ultrasound Doppler was performed using the premium EPIQ 7 US system (Philips, Netherlands). The following indices of carotid stiffness and atherosclerosis were assessed:

#### Carotid intima-media thickness (cIMT)

IMT is defined as the distance between the media–adventitia interface and the lumen–intima interface. Measurements were performed bilaterally at the far wall of the common carotid artery (CCA) 1.0 cm proximal to the carotid bifurcation. The measurement was for a length of 10 mm, which consisted of 150 points of measurements, on the far wall of the common carotid artery on both sides, as described in previous studies [16]. Plaque was defined according to the Mannheim consensus [17], in which a plaque was diagnosed when the vessel wall thickness was > 1.5 mm or when the vessel wall appeared to be ≥ 0.5 mm or 50% thicker than the surrounding wall. The mean value of computer-based points was used. For each individual, cIMT was determined as the average of 3 measurements for each artery, as was automatically computed by the QLAB software (Philips, Netherlands).

#### 3-D Carotid plaque volume

Patients with detectable plaques in the carotid artery went through plaque volume analysis. In standard optimized mode, using the mechanic volumetric VL13-5 broadband linear array transducer, 3D plaque scanning volume data were obtained automatically. For each volume approximately 250 single transverse images (frames) were obtained with an interval of 0.15 mm. Plaque volume was automatically calculated using the Vascular Plaque Quantification (VPQ) module (QLAB software), after selecting the beginning and ending frame and selecting at least one key frame within the plaque region.

#### Carotid Distensibility

Following static B-mode real-time imaging from a longitudinal section of the CCA after 5 min of rest, dynamic CINE looped M-mode images of consecutive cardiac cycles were stored for later offline analysis. Distensibility was assessed using the distension of both CCAs, measuring the change in diameter in systole relative to diastolic during the cardiac cycle. The vessel lumen diameter was assessed from the near wall to the far wall of the CCA. The maximal systolic lumen diameter was determined visually and from the R-wave of the ECG-recording and the minimal lumen diameter was used for the diastolic diameter. The end-diastolic diameter (Dd), the absolute stroke change in diameter during systole (∆A), and the relative stroke change in diameter (∆A/Dd) were computed as the mean of 10 cardiac cycles of one successive recording. Blood pressure was measured before and after the measurement session and pulse pressure (∆P) was defined as the difference between the systolic and diastolic blood pressure. The cross-sectional arterial wall distensibility coefficient was calculated according to the following equation:

$${\text{Crosssectional distensibility coefficient }}\left( {\text{DC}} \right)\, = \,1000*\frac{\Delta A}{A}*\Delta P\;\left[ {{\text{kPa}}^{ - 1} } \right]$$

#### Carotid Elastography

In the B-mode display, a midsection of straight CCA in longitudinal plane is chosen. The shear wave elastographic mode was activated to show paired images of B-mode and elastography at the same time. The probe was handed with standardized pressure in the 2nd–4th quintile of the linear pressure scale, as seen using standardized real-time measurement displayed on a linear scale. Elastographic images are displayed with different color mapping for the softest, intermediate and hardest components, according to the different levels of strain. On a representative static image, the relative strain ratio (SR), between blood to carotid arterial wall were measured. The first region of interest (ROI) for the arterial wall strain was manually placed at the midpoint of posterior wall of displayed carotid artery. The second ROI for the blood strain was placed at the center of arterial lumen. SR was calculated automatically by dividing strain value of the blood by that of carotid arterial wall, using the QLAB software. Measurement were performed during 10 heart beats and an average of 3 images as described above, preferably consecutive, were used as the elastography index.

### Covariates

Data on risk factors and possible confounders were obtained using the Maccabi Diabetes Registry and data collection during the baseline visit of the IDCD cohort. Variables available through the Maccabi Diabetes Registry were computed as the average of all the measurements done in Maccabi since the subject entered the Diabetes Registry. Variables extracted from Maccabi Diabetes Registry included time in the diabetes registry (a proxy of duration of diabetes) [18], BMI, HbA1c total, HDL and LDL cholesterol, triglycerides, CRP, eGFR, diabetes treatment and smoking. All blood measurements obtained from Maccabi were analyzed at a central lab. BMI was calculated using height and weight measured at the physician office. During the baseline visit of the IDCD study blood samples were obtained to evaluate CRP. Blood pressure was measured during the carotid artery US examination.

### HbA1c

HbA1c was measured with standard methods of high-performance liquid chromatography using an ion exchange column. Participants were assessed under fasting conditions approximately annually at the MHS.

### Crp

C-reactive protein (mg/L) was measured from plasma, using the ADVIA 1650 Chemistry System with a CRP latex reagent.

### Statistical methods

All variables were reviewed for abnormal values, to assess skewedness and outliers. Characteristics of study participants between the 3 trajectory groups were compared using independent-samples *T* test, Wilcoxon rank-test and ANOVA, as appropriate for continuous variables, and χ^2^ test for categorical variables.

The outcomes (cIMT, distensibility coefficient and elastography strain ratio) were defined as the average of the measurements in the right and the left CCA. Carotid plaque volume total burden was defined as the sum of the plaque volume in the right and the left. Carotid plaque was categorized into 4 groups: no plaque group, and tertiles of the plaque volume: small plaque (volume ≤ 122 mm^3)^ medium plaque (volume 122.1–271 mm^3^) and large plaque (volume > 271 mm^3^). Continuous variables were categorized for worst quartiles (for cIMT > 0.9 mm, for carotid elastography SR < 0.925 and for carotid distensibility DC < 13.03 10^3^/Pa^−1^). Linear regression was used to estimate the association (β) and 95% confidence interval (CI) between the outcomes and mean BMI as a continuous dependent variable or BMI trajectory group. Logistic regression was used to estimate the odds ratio (OR) and 95% CI for the association between the outcomes and mean BMI as a continuous dependent variable or BMI trajectory group. Primary covariates in all analyses were age and gender as they are strongly associated with both predictors and outcomes. Secondary covariates were LDL cholesterol, triglycerides, CRP, eGFR, diabetes treatment, duration of diabetes and smoking. Diabetes treatment was defined as the treatment type: no medication, oral medication, insulin, oral and insulin. For the regression models estimating the association with carotid distensibility, blood pressure was not used as a covariate since it is part of the DC equation. Statistical analysis was performed using SPSS software v24.

#### Calculation of trajectories

The Maccabi Diabetes Registry has BMI registered since 1998 for patients undergoing their annual visits. Trajectories of BMI were identified using a SAS macro (PROC TRAJ), which applies a multinomial modeling strategy to identify relatively homogenous clusters of developmental trajectories within a sample population. Trajectory parameters are derived by latent class analysis using maximum likelihood estimation. In particular, the distinctive trajectories of BMI were derived by modeling BMI as a function of the number of follow-up years in the Diabetes Registry prior to the start date of IDCD (defined as the intercept) with the adjustment of IDCD baseline age and gender. Distinct time points were created for each follow-up visit observed. The number of trajectories and degree of curvature were determined using the guidelines suggested by Jones et al. [19]. For the study population mean number of BMI measurements per subject was 12.17 ± 13.1, with 14 subjects that had only 1 measurement of BMI. For subjects with 1 measurement, trajectory was calculated using the single measurement. Three trajectories were identified with linear, quadratic and cubic curves corresponding to normal, overweight and obese BMI groups, respectively. The output of PROC TRAJ includes the equations for the different trajectories along with the assignment of each patient to one of the trajectory groups.

## Results

**Fig. 2:**
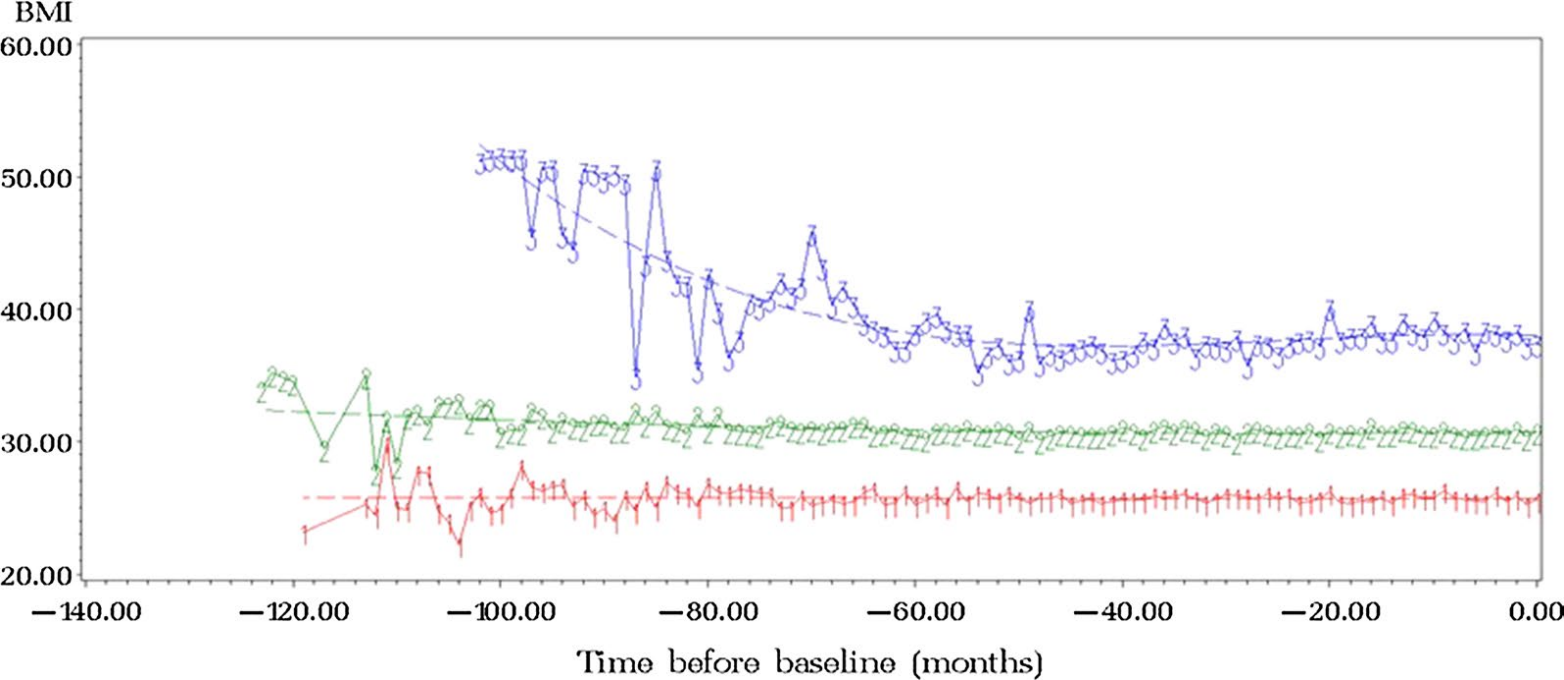
BMI groups. Three types of trends of BMI over time: 1-“normal”, 2-”overweight” and 3-”obese”

Subjects were 42% female, mean age of 76.4 ± 4.4 years, mean HbA1c of 6.7 ± 0.7% (50 ± 7.7 mmol/mol). Mean BMI was 29.19 ± 4.37 kg/m^2^, consistent with an overweight diabetic sample. Diabetes medication consumption according to medication groups at baseline was: 71% metformin, 30% sulfonylurea, 14% meglitinides, 8% insulin, 6% TZDs and 1% GLP-1 agonists. Three types of trends of BMI over time were observed: “normal” (44%, n = 185), “overweight” (44.8%, n = 188), and “obese” (11.2%, n = 47). The normal and overweight trajectories, represented as 1 and 2 respectively on the graph in Fig. 2, were stable over time, while the obese trajectory, represented as 3 on the graph in Fig. 2, had a tendency to decline but remained stable at the obese levels.

The correlation between the different carotid atherosclerosis characteristics was estimated using the pearson ρ. The correlation between carotid distensibility and carotid elastography was low (ρ^2^ = 0.12, p = 0.022). There was no correlation between cIMT with distensibility (ρ^2^ = − 0.01, p = 0.848) or elastography (ρ^2^ = − 0.043, p = 0.396). Large carotid plaque volume was correlated with more cIMT (ρ^2^ = 0.160, p = 0.001) and poorer carotid elastography (ρ^2^ = − 0.131, p = 0.007), but not with distensibility (ρ^2^ = 0.046, p = 0.384).

**Fig. 3:**
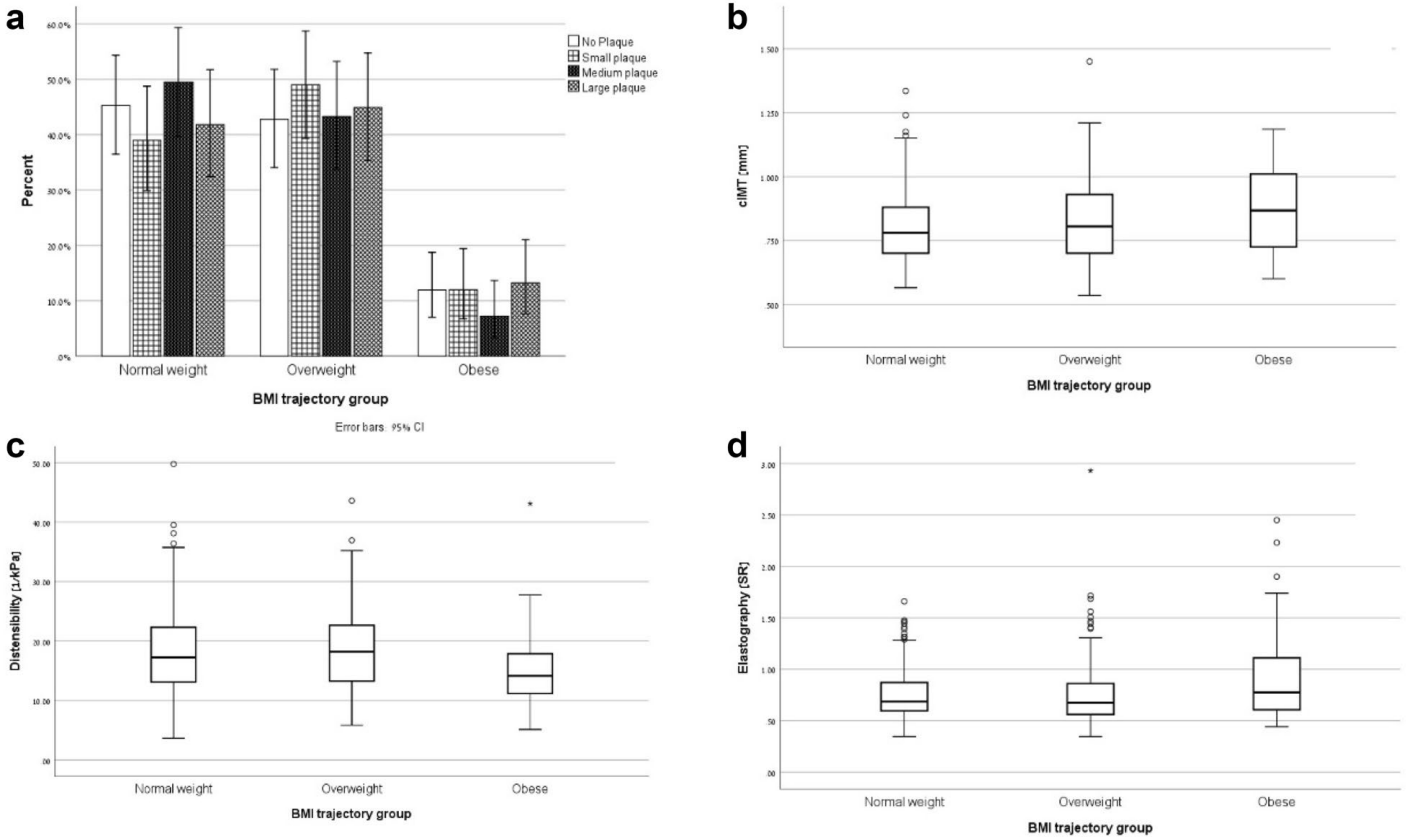
Distribution of the carotid atherosclerosis measurements among the 3 trajectory groups: **a** plaque volume prevalence, **b** cIMT, **c** Distensibility and **d** elastogreaphy

Baseline characteristics by BMI trajectory groups are summarized in Table 1. BMI trajectory groups differed in age, triglycerides, HDL cholesterol, systolic and diastolic blood pressure but not in years of diabetes, mean HbA1c, LDL, eGFR, diabetes medication and smoking (Table 1). Indices of carotid stiffness and atherosclerosis by BMI trajectory groups are depicted in Fig. 3.

**Table 1:** Distribution of risk factors among the different BMI trajectory groups

	Normal weight (n = 185)	Overweight (n = 188)	Obese (n = 47)	p
Gender				
Male	114 (61.6%)	110 (58.5%)	24 (51.1%)	0.413
Female	71 (38.4%)	78 (41.5%)	23 (48.9%)	
Age [years]	76.99 ± 4.33	75.95 ± 4.41	75.87 ± 3.88	0.046
Years diagnosed	9.54 ± 4.54	9.71 ± 4.49	9.21 ± 4.14	0.775
Mean HbA1c	6.62 ± 0.69	6.71 ± 0.73	6.75 ± 0.8	0.334
[%] (mmol/mol)	48.9 ± 4.4	49.8 ± 5.6	50.3 ± 6.4	
Triglycerides [mg/dL]	143.45 ± 62.61	163.25 ± 77.99	171.39 ± 62.98	0.006
LDL cholesterol [mg/dL]	100.98 ± 20.72	101.35 ± 19.68	97.06 ± 20.45	0.417
HDL cholesterol [mg/dL]	50.21 ± 11.57	47.36 ± 10.6	45.72 ± 9.72	0.009
CRP [µgr/ml]	0.88 ± 2.07	1.02 ± 1.24	1.36 ± 1.21	0.208
Systolic BP [mmHg]	141.42 ± 22.12	142.7 ± 20.09	153.31 ± 25.79	0.004
Diastolic BP [mmHg]	70.64 ± 9.79	74.32 ± 9.11	76.26 ± 14.38	0.001
eGFR [ml^−1^ *min^−1^ *1.73 m^−2^]				
≤ 45	5 (2.7%)	2 (1.1%)	1 (2.1%)	0.594
45–60	29 (15.7%)	37 (19.9%)	12 (25.5%)	
≥ 60	151 (81.6%)	147 (79%)	34 (72.3%)	
Diabetic medications				
No medication	28 (56.0%)	19 (12.3%)	3 (0.9%)	0.498
Oral medication	115 (73.7%)	121 (78.1%)	29 (80.6%)	
Insulin	13 (8.3%)	15 (9.7%)	4 (12.5%)	
Smoking				
Never smoked	80 (43.7%)	65 (35.5%)	14 (31.1%)	0.269
Smoked in the past	85 (46.4%)	93 (50.8%)	27 (60.0%)	
Smoking	18 (9.8%)	25 (13.7%)	4 (8.9%)

Association of mean BMI and BMI trajectories (“normal”, “overweight” and “obese”) with indices of carotid stiffness and atherosclerosis estimated by linear regression are summarized in Table 3. Participants from the obese trajectory group had higher carotid elastography strain ratios, lower distensibility coefficients and higher cIMT than participants in the normal trajectory group (Table 2).

**Table 2:** Distribution of atherosclerosis markers between the BMI trajectory groups

	Normal weight	Overweight	Obese	p
Carotid plaque volume	(n = 181)	(n = 185)	(n = 46)	0.703
No plaque	53 (29.3%)	50 (27.0%)	14 (30.4%)	
Small plaque	39 (21.5%)	49 (49.0%)	12 (26.1%)	
Medium plaque	48 (26.5%)	42 (22.7%)	7 (15.2%)	
Large plaque	41 (22.7%)	44 (23.8%)	13 (28.3%)	
Carotid IMT [mm] (n = 407)	0.801 ± 0.14 (n = 166)	0.819 ± 0.16 (n = 169)	0.868 ± 0.16 (n = 36)	0.057
Carotid Distensibility [kPa^−1^] (n = 376)	18.39 ± 7.46 (n = 154)	18.65 ± 7.16 (n = 154)	15.02 ± 6.6 (n = 41)	0.015
Carotid Elastography [SR] (n = 432)	0.757 ± 0.26 (n = 178)	0.757 ± 0.31 (n = 179)	0.932 ± 0.46 (n = 42)	0.003

**Table 3:** Association of mean BMI and BMI trajectories (“normal”, “overweight” and “obese”) with indices of carotid stiffness and atherosclerosis estimated by linear regression

Dependent variable	β	CI	p-value
Distensibility coefficient^a^			
Mean BMI	− 0.275	[− 0.469, − 0.082]	0.005
Normal weight	0		
Overweight	0.170	[− 1.584, 1.924]	0.849
Obese	− 3.078	[− 5.974, − 0.182]	0.037
Elastography strain ratio			
Mean BMI	0.006	[− 0.002, 0.015]	0.139
Normal weight	0		
Overweight	− 0.014	[− 0.086, 0.057]	0.698
Obese	0.181	[0.058, 0.304]	0.004
Cimt			
Mean BMI	0.005	[0.001, 0.009]	0.026
Normal weight	0		
Overweight	0.033	[− 0.002, 0.069]	0.068
Obese	0.095	[0.030, 0.160]	0.004
Plaque volume group^b^			
Mean BMI	-0.005	[− 0.053, 0.044]	0.843
Normal weight	CG		
Overweight	0.146	[− 0.286, 0.577]	0.508
Obese	0.066	[− 0.661, 0.793]	0.858

Models were adjusted to age, gender, diabetes parameters (years of diabetes, medication and HbA1c), blood lipids, CRP, blood pressure, eGFR and smoking status

^a^Distensibility was not adjusted to blood pressure; ^b^Plaque volume association was estimated by ordinal regression for carotid plaque volume group

Association of mean BMI and BMI trajectories with cutoffs for indices of carotid stiffness and atherosclerosis estimated by logistic regressions are summarized in Table 4. Participants from the obese trajectory group had a 2.79-fold increased odds for impaired distensibility, 5.28-fold increased odds for thickened cIMT and 8.46-fold increased odds for large plaque volume compared with the normal BMI trajectory group.

**Table 4:** Risk ratio estimation of mean BMI and BMI trajectories (“normal”, “overweight” and “obese”) with indices of carotid stiffness and atherosclerosis estimated by logistic regression

Dependent variable	OR	CI	p-value
Distensibility coefficient^a^			
Mean BMI	1.080	[1.011, 1.153]	0.022
Normal weight	CG		
Overweight	1.083	[0.565, 2.047]	0.810
Obese	2.790	[1.087, 7.158]	0.033
Elastography strain ratio			
Mean BMI	1.035	[0.972, 1.103]	0.278
Normal weight	CG		
Overweight	1.138	[0.630, 2.065]	0.668
Obese	1.965	[0.801, 4.818]	0.140
cIMT			
Mean BMI	1.126	[1.051, 1.206]	0.001
Normal weight	CG		
Overweight	2.294	[1.264, 4.164]	0.006
Obese	5.277	[2.013, 13.838]	0.001
Carotid plaque volume			
Mean BMI	0.995	[0.941, 1.052]	0.866
Normal weight	CG		
Overweight	2.205	[0.967, 5.027]	0.060
Obese	8.456	[1.559, 45.863]	0.013

Models were adjusted to age, gender, diabetes parameters (years of diabetes, medication and HbA1c), CRP, blood lipids, blood pressure, eGFR and smoking status

^a^Distensibility was not adjusted to blood pressure

An additional sub-study (n = 36) for the repeatability and reliability of carotid plaque volume measurement was conducted, demonstrating good intra-observer (ICC = 0.92, p < 0.001) and inter-observer (ICC = 0.90, p < 0.001) agreement. For post processing of plaque volume there was excellent intra-observer (ICC = 0.97, p < 0.001) and inter-observer (ICC = 0.84, p < 0.001) agreement.

## Discussion

In this study we have found that among elderly cognitively normal patients with type 2 diabetes, that longitudinal measurements of adiposity are associated with indices of carotid stiffness and atherosclerotic plaque volume. Adjustment of the models for several relevant cardiovascular and sociodemographic variables did not attenuate these associations.

This study provides new evidence in several levels. The study focuses on an elderly diabetic population on which there is scarce evidence on relationships of obesity with measures of carotid stiffness and atherosclerotic plaque volume, despite the biological plausibility of this association. Using a broad battery, we have found that adiposity is associated differentially with carotid indices, suggesting that adiposity affects some carotid features more than others. Finally, we have used long-term data of BMI that span approximately 26 years and presented both as mean of all measurements and trajectories of BMI.

In this study we have found that BMI is associated with carotid artery stiffening as indicated by carotid distensibility and elastography, in addition to the association with carotid atherosclerosis parameters as indicated by cIMT and carotid plaque volume. Arteries are known to stiffen in healthy aging and with atherosclerosis, diabetes, hypertension and obesity [20]. Stiffening of the carotid arteries is associated with higher risk for stroke [21]  and is predictive of white matter hyperintensity volume and total brain volume [22]. It has been suggested that stiffening of the carotid artery increases the mechanical force on existing plaque and as a result increases the risk for rupture of existing plaques [23].

Our data provide additional support to the growing evidence on the association of obesity with impaired vascular health. Impaired vascular health in the carotid artery wall was observed as early as in hypertensive children [24], healthy pre- and early pubescent children [25], adolescents [9, 26], and physically inactive adults office workers [6]. In a case control study among adults, obesity was associated with cIMT (but not distensibility) [7, 27]. Increased cIMT and decreased distensibility were observed among adult men [28] and women [29, 30]. The long-term effects of increased childhood BMI on adulthood carotid atherosclerosis was examined in several studies. In two cohort studies BMI trajectories that were identified from childhood to adulthood increased the risk for impaired cIMT [31, 32]. An additional study found an association of BMI in childhood, young adulthood and cumulative risk with impaired cIMT among healthy population [33]. In addition, obesity was found to be the most influencing risk factor for unstable carotid plaque among men under 70 [34]. A research looking at CVD risk trajectories from childhood to adulthood found that many of those who had consistently high BMIs during the life-course were better protected from carotid intima-media thickening if they were physically active [35]. Furthermore, in additional studies, no association was found between BMI and carotid atherosclerosis in non-diabetic [36] as well as diabetic patients [5, 37]. We are not aware of any published evidence on the association of carotid elastography and BMI.

An additional aspect is variability in BMI over time, which has been associated with other old age outcomes such as dementia [46]. BMI variability over time in addition to the trajectory of BMI over time, might affect carotid atherosclerosis. We have examined the associations of standard deviation of all BMI measures available for each subject with the carotid measures and found no significant associations (data not shown) suggesting that in this sample of older adults, variability in BMI does not predict carotid vascular health.

Several underlying biological mechanisms may link BMI with impaired vascular health. In our study obese patients were younger, yet had higher triglycerides and blood pressure, and lower HDL cholesterol, all independent risk factors for atherosclerosis in general and carotid artery atherosclerosis in particular [38]. However, adjusting for these risk factors did not attenuate the BMI-carotid associations suggesting involvement of other mechanisms. Second, adipocytokines, i.e. fat-related inflammatory markers such as IL-6 and leptin, play an important role in atherosclerosis including initial activation of endothelial cells, through atherosclerotic progression and, ultimately, its final complication, thrombosis [39]. As was shown in a cohort of elderly patients with type 2 diabetes, adipose tissue-derived inflammatory factors resistin, vaspin and visfatin were associated with pathogenesis of carotid atherosclerosis [40]. Third, visceral abdominal fat has a direct circulatory connection to the liver. Excessive release of free fatty acids from visceral adipose tissue directly in the portal circulation might lead to insulin resistance and hyperlipidemia, both established risk factors for CVD [41].

Our study has several limitations. The study is conducted on elderly non-demented type 2 diabetes patients which may reflect a population of “survivors”, so subjects who were eligible for this study were those who were not demented after 2 IDCD follow up visits over approximately four years. The IDCD study focuses on older adults with type 2 diabetes and thus conclusions from this study cannot be extrapolated to non-diabetic populations. We have only cross-sectional carotid artery data so reverse causality cannot be ruled out. However, the BMI trajectories spanned 26 years suggesting that long-term obesity may be a predictor of carotid disease.

An additional limitation is that our measure of adiposity was BMI, so we could not directly address aspects of body composition such as visceral fat or fat distribution [42–44]. There is, however, evidence that in children combination of total and central measures of fat does not improve the prediction of increased cIMT, and a simple surrogate measures of fatness such as BMI can be used [45].

Strengths of our study include its relatively large-scale, the in depth carotid assessments including novel indices of impaired vascular health, a directly measured (rather than self-reported) diabetes diagnosis, and an exquisite characterization of long-term covariates and of BMI trajectories derived from the Maccabi Diabetes Registry data.

## Conclusion

Our results suggest that mean BMI and BMI trajectories of obesity are associated with subclinical atherosclerosis and impaired vascular health among elderly non demented type 2 diabetes patients. Further studies should assess the effect of weight reduction on subclinical carotid atherosclerosis, and whether the associations of BMI with macro-vascular complications of diabetes such as stroke are mediated by carotid atherosclerosis.

## Data Availability

The datasets used and/or analysed during the current study are available from the corresponding author on reasonable request.

## Abbreviations

BMI: Body mass index
CCA: Common carotid artery
CI: Confidence interval
cIMT: Carotid intima media thickness
HMO: Health maintenance organization
IDCD: Israel diabetes and cognitive decline
MHS: Maccabi healthcare services
OR: Odds ratio
SR: Strain ration
VPQ: Vascular plaque quantification
ROI: Region of interest
